# State of the Art in Crystallization of LiNbO_3_ and Their Applications

**DOI:** 10.3390/molecules26227044

**Published:** 2021-11-22

**Authors:** Kunfeng Chen, Yunzhong Zhu, Zhihua Liu, Dongfeng Xue

**Affiliations:** 1 State Key Laboratory of Crystal Materials, Institute of Crystal Materials, Shandong University, Jinan 250100, China; Kunfeng.Chen@sdu.edu.cn; 2 Sino French Institute of Nuclear Engineering and Technology, Sun Yat-sen University, Zhuhai 519082, China; zhuyzh7@mail.sysu.edu.cn (Y.Z.); liuzhh229@mail.sysu.edu.cn (Z.L.); 3 Multiscale Crystal Materials Research Center, Shenzhen Institute of Advanced Technology, Chinese Academy of Sciences, Shenzhen 518055, China

**Keywords:** LiNbO_3_, crystal growth, piezoelectric property, optical property

## Abstract

Lithium niobate (LiNbO_3_) crystals are important dielectric and ferroelectric materials, which are widely used in acoustics, optic, and optoelectrical devices. The physical and chemical properties of LiNbO_3_ are dependent on microstructures, defects, compositions, and dimensions. In this review, we first discussed the crystal and defect structures of LiNbO_3_, then the crystallization of LiNbO_3_ single crystal, and the measuring methods of Li content were introduced to reveal reason of growing congruent LiNbO_3_ and variable Li/Nb ratios. Afterwards, this review provides a summary about traditional and non-traditional applications of LiNbO_3_ crystals. The development of rare earth doped LiNbO_3_ used in illumination, and fluorescence temperature sensing was reviewed. In addition to radio-frequency applications, surface acoustic wave devices applied in high temperature sensor and solid-state physics were discussed. Thanks to its properties of spontaneous ferroelectric polarization, and high chemical stability, LiNbO_3_ crystals showed enhanced performances in photoelectric detection, electrocatalysis, and battery. Furthermore, domain engineering, memristors, sensors, and harvesters with the use of LiNbO_3_ crystals were formulated. The review is concluded with an outlook of challenges and potential payoff for finding novel LiNbO_3_ applications.

## 1. Introduction

Due to its piezoelectric, ferroelectric, nonlinear optics, and pyroelectric properties, LiNbO_3_ crystal has found its wide applications in surface acoustic wave (SAW) devices, optical waveguides, optical modulators, and second-harmonic generators (SHG) [1,2,3]. LiNbO_3_ crystallized as R3c space group below Curie temperature shows spontaneous polarization that leads to its ferroelectric and piezoelectric properties [4,5]. Physical and chemical characteristics of LiNbO_3_ are mainly determined by Li/Nb ratio, impurity cations, vacancies in a cation sublattice [6,7,8]. Different sizes of LiNbO_3_ ranging from nanoscale and microscale to bulk size have been synthesized by solid state method, hydrothermal/solvothermal method, Czochralski (Cz) growth method, etc. Most basic and applied studies of LiNbO_3_ focus on its bulk single crystal [9,10,11]. The Cz growth method is the current mainstream technology for growing high quality bulk single crystal LiNbO_3_. Generally, congruent LiNbO_3_ melt composition is used in Cz method, leading to the growth of congruent LiNbO_3_ (CLN) crystal, which suffers from Li deficient (about 48.6 mol. % Li_2_O) [12,13]. However, The CLN crystal contains a high concentration of intrinsic defects in the form of Nb anti-sites (Nb_Li_^5+^) and Li vacancies (V_Li_^−^) that limit its scope for optical applications [14,15].

**Figure 1:**
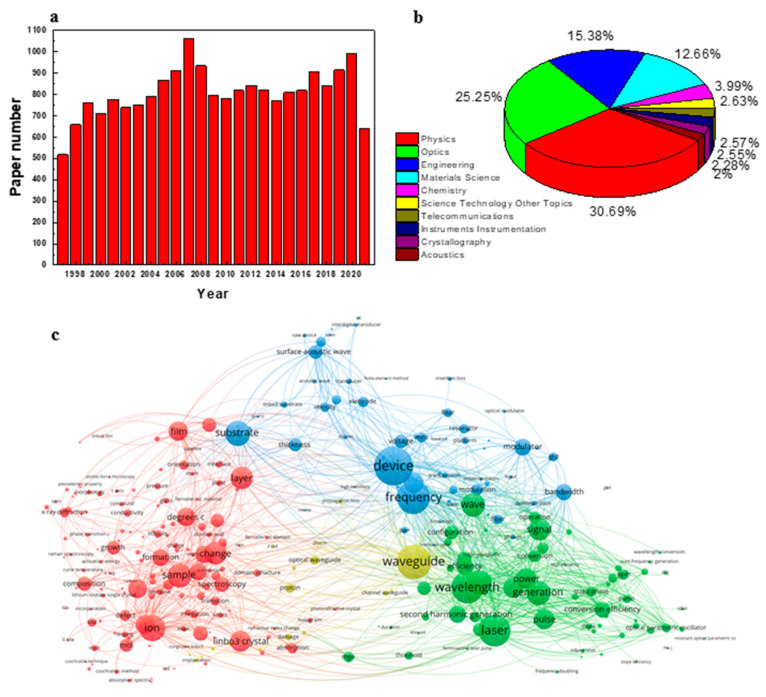
(**a**) Growth of scientific publications referring to LiNbO_3_ over 1997–2021. (**b**) Pie chart of research areas about LiNbO_3_. (**c**) Hot research directions of LiNbO_3_ crystals. The results of a search performed using “Topic = LiNbO_3_ or lithium niobate” on Web of science.

To quantitatively display the development trends of LiNbO_3_ researching field, the Web of Science database was used to track the number of publications between 1997 and 2021 (Figure 1a). The results of a search performed using “Topic = LiNbO_3_ or lithium niobate”. Recently, the growth trend of publications about LiNbO_3_ can be found from 2018 (Figure 1a). Three top research areas about LiNbO_3_ are physics, optics, and engineering (Figure 1b), in this review we also focus on discussion about the application of LiNbO_3_ in physics, optics, and engineering fields. According to visualization analysis, Figure 1c shows hot research directions of LiNbO_3_ materials and their relationships. For application areas, waveguide, laser, SAW, SHG devices are the most studied directions. For LiNbO_3_ crystallization, researchers focus on the areas of growth, doping, defect, etc. Some characterization methods also received more study attention, for example, spectroscopy, X-ray diffraction, Raman, which are important to elucidate their structure–performance relationships.

According to research highlights of LiNbO_3_, in this review, we focus our discussions on structure, growth, characterizations, and advanced applications of LiNbO_3_. In Section 2, crystal and defect structures of LiNbO_3_ are focused. The growth methods of Cz are discussed in Section 3. Different characterizations to determine Li content are analyzed in Section 4. In Section 5, the advanced applications with the utilization of LiNbO_3_’s optical, piezoelectric, ferroelectric, nonlinear optics, and pyroelectric properties, are reviewed. Finally, conclusions and an outlook are presented in Section 6.

## 2. Crystal and Defect Structures of LiNbO_3_

**Figure 2:**
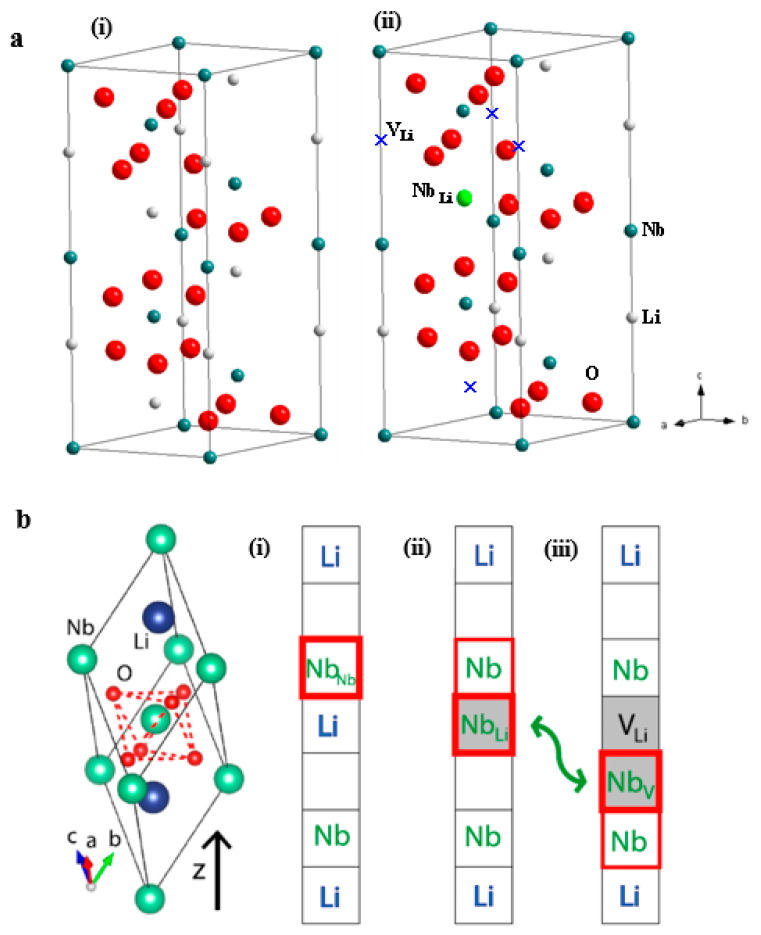
(**a**) Crystal structures of stoichiometric LiNbO_3_ (i) and congruent LN with anti-site Nb_Li_^4+^ and V_Li_^−^ defects (ii) [22]; (**b**) Free and defect-bound (bi)polarons in LiNbO_3_ [17].

Crystal structure of LiNbO_3_ can be described as hexagonal unit cells (Figure 2a) or rhombohedral unit cells [16,17]. In stoichiometric LiNbO_3_, along c row direction, the O octahedral interstitials are filled by Li ions (one-third), Nb ions (one-third), and empty (one-third), forming –Li–Nb–▯–Li–Nb– sequence [18,19,20]. Much experimental and simulation effort have been made in the past in order to understand the defect structure in LN crystal [8]. Several defect models have been constructed—i.e., oxygen vacancy model, niobium vacancy model ([Li_1−5x_Nb_5x_][Nb_1−4x_V_4x_]O_3_), and lithium vacancy model ([Li_1−5x_V_4x_Nb_x_]NbO_3_) [18,19,20]. Congruent LiNbO_3_ crystals were grown with LiCO_3_ and Nb_2_O_5_ as starting materials, which contain a high concentration of Nb anti-sites (Nb_Li_^4+^) and Li vacancies (V_Li_^−^) (Figure 2a(ii)) [21]. Owing to atomic radius differences between Nb and Li, it forbids Li replacement in a Nb site. Thus, the composition deviates from stoichiometric only toward the Nb-rich side [22,23]. The Li vacancy model is mostly accepted nowadays thanks to a great number of investigations, some of them very important and performed in the 1990s. This is given in detail in [8]. Since these defects are charged, further defects with counter charges are required in order to guarantee overall charge neutrality [23]. Thus, for energetic reasons, complex ionic complexes and spaced clusters are present as shown in Figure 2b [17]. However, debate still prevails on the available models on defect clusters. The understanding and control of LiNbO_3_ intrinsic and extrinsic defects during crystallization and operational process is important for specific applications.

## 3. Crystallization of LiNbO_3_

**Figure 3:**
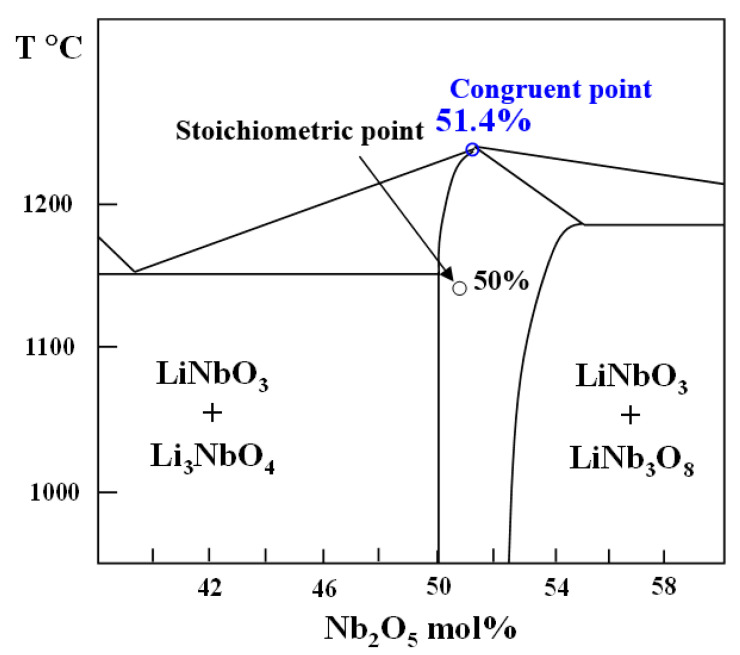
Schematic equilibrium phase diagram of binary system of Li_2_O and Nb_2_O_5_ m in the vicinity of LiNbO_3_.

According to binary phase diagram, LiNbO_3_ has a large solid solution range, which can exist and be stable on Li composition from 46.5 mol% to 50 mol% (Figure 3). The liquid–solid curve reveals a diffuse maximum at approximately 48.6% Li_2_O [24]. With exceeding composition range, the secondary LiNb_3_O_8_ and Li_3_NbO_4_ phases can be created. The binary phase diagram can be determined by measuring XRD of different samples along solid lines. However, it is also needed to probe precise composition range, because LiNbO_3_’s bulk properties are composition dependent [25].

LiNbO_3_ polycrystalline can be grown by solid-state reaction, sol–gel, hydrothermal, vapor phase methods. The crystallization method of LiNbO_3_ single crystal includes Cz, Bridgeman, high-temperature top-seeded solution growth. Cz method is the current mainstream technology for growing bulk LiNbO_3_ single crystal [26,27,28]. With LiNbO_3_ polycrystalline as starting materials, the Cz crystal growth is often controlled by the pulling/rotation rate and heater power [29,30]. The growth of LiNbO_3_ crystal was affected by various factors together, such as the ratio of raw materials, quality of seed crystal, temperature gradient, growth parameters, etc. [24]. In reality, the Li evaporation at high temperature is hard to be eliminated, which results in the segregation of Li content inside the as-grown crystal. Congruent LiNbO_3_ with good compositional uniformity can be formed with Li content can range from 47 to 50 mol%. Nearly stoichiometric LiNbO_3_ composition can be achieved by more elaborate growth processes.

A slower pulling rate is helpful to obtain a crystal with less internal stress and high quality. Table 1 shows pulling rate and rotation rate [26,27,28,29,30]. Recently, 6-inch LiNbO_3_ crystals have been grown with a rotation rate of 5~10 rpm, and the pulling rate of 1–2 mm/h [26]. The obtained 6-inch LiNbO_3_ crystal shows good homogeneity with the absolute deviation of Curie temperature ≤1.3 °C. In addition, fast growth rate can lead to low-cost LiNbO_3_ crystal, which is important for industry production. Thus, under the premise of ensuring quality, fast pulling rate is also demanded.

## 4. Composition Characterizations of LiNbO_3_

The performances of LiNbO_3_ are most depend upon their chemical composition. Therefore, the development of the precise analysis method to detect the chemical composition (Li content) of LiNbO_3_ is very important. Table 2 shows available testing methods for determine Li content of LiNbO_3_, for example, X-ray diffraction (XRD), Raman spectroscopy (RS), UV–vis diffuse reflectance (DR), and differential thermal analysis (DTA) [31,32,33,34,35,36].

In Raman spectroscopy, the Li content can be calculated according to the linewidth (Γ) at 876 cm^−1^ [37,38,39].

C_Li_ = 53.29–0.1837Γ (1)

The Li content of LiNbO_3_ can be also calculated via measuring Curie temperature

C_Li_ = 17.37 + 0.02725T_c_ (2)

where T_c_ is Curie temperature in °C. Curie temperature is the temperature at which LiNbO_3_ tends to lose its ferroelectric properties. When use above reported characterizations, the applicability and calibration method need to be concerned. Some indirect optical and non-optical methods for the determination of the chemical composition of LN single crystals can be referred to [8].

## 5. Advanced Applications of LiNbO_3_

### 5.1. Optical Applications

LiNbO_3_ presents remarkable properties including low cut-off phonon energy, high stability in high temperature, acid and alkali proof, which becomes an attractive luminescence carrier among all the oxide matrixes [40,41]. Therefore, lanthanide-based LiNbO_3_ phosphors are widely investigated for the application fields, especially in illumination, LED, and fluorescence temperature sensing.

#### 5.1.1. Illumination

The lanthanide-based illumination mainly relies on inner 4*f* transitions to achieve visible luminescence via Stokes and anti-Stokes processes assigned to downshifting and upconversion emission, respectively. To be specific, the downshifting emission refers to tailoring process wherein high energy photons are converted into low energy photons; while the upconversion process is an inverse process. On the bases of frequency converted mechanism, the lanthanide-based phosphors are widely applied in white-light luminescence. However, the most concerned problem is the quantum yield of the luminescence process, since the selective rules greatly hinder the 4*f* transition probability. In recent years, remarkable progress has been achieved in the promotion of luminescence intensity in aspects of concentration optimization, local crystal field symmetry tailoring, energy transfer promotion, and local surface plasmon resonance (LSPR).

**Figure 4:**
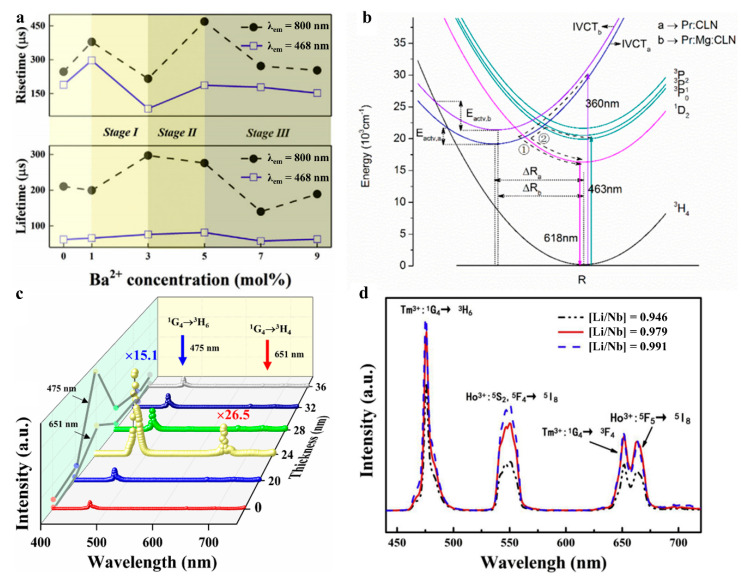
(**a**) The dependence of lifetime and rise time upon Ba^2+^ concentration with 468 and 800 nm [41]; (**b**) The configurational coordinate diagram of Pr^3+^:CLN and Pr^3+^/Mg^2+^:CLN, depicting the light emitting mechanism based on the intervalence charge transfer [42]; (**c**) The UC spectra of Au-coated Tm^3+^/Yb^3+^/LN wafers in different film thickness [43]; (**d**) Upconversion emission spectra of Ho^3+^/Yb^3+^/Tm^3+^:LiNbO_3_ single crystals with different [Li]/[Nb] ratios ([Li]/[Nb] = 0.946, 0.979 and 0.991) under 980 nm excitation at room temperature [44].

Yang and his co-workers have grown a series of Sm^3+^ doped LiNbO_3_ single crystal from 0.2 to 2 mol%, and pointed out that the luminescence intensity achieves the maximum value in 1 mol% Sm^3+^ sample with 409 nm irradiation [40]. Theoretically simulation is applied to explain the luminescence mechanism based on Judd–Ofelt theory, suggesting the existing ${Sm}_{Li}^{2+}$-${Sm}_{Nb}^{2-}$ centers plays an important role in the luminescence. Similarly, Liu and co-workers have promoted the energy transfer rate between the luminescence center of Tm^3+^ and Yb^3+^ via incorporation of Ba^2+^ in Tm^3+^/Yb^3+^:LN polycrystal to form ${Tm}_{Li}^{2+}$-${Yb}_{Nb}^{2-}$ centers [41]. As shown in Figure 4a, the rise times associated with the energy transfer rate in a series of Ba^2+^/Tm^3+^/Yb^3+^:LN are illustrated, accompanying with the schematic diagrams for corresponding cation site occupancy construction. On the other hand, metal dopant could optimize the energy transfer process as well. Long and co-workers have enhanced the red emission in Pr:LN single crystal via co-doping with Mg^2+^ [42]. The incorporation of Mg^2+^ promotes the electronic population on the Pr^3+^(^1^D_2_) level via intervalence charge transfer with 360 nm irradiation as shown in Figure 4b, which further increases the 618 nm emission. Optimizing the energy transfer process could promote the energy efficiency and benefit the electron population on the emission level. To further improve the luminescence intensity, LSPR technique is applied via a nano-scale Au coating and largely promotes the overall luminescence intensity on Tm^3+^/Yb^3+^:LN single crystal. Liu et al. have proposed of using nano-scale Au-coating to enhance the luminescence intensity of an as-grown single crystal wafer, wherein the enhancement factor presents strong thickness dependence, as presented in Figure 4c [43]. This mechanism is revealed in two aspects: the promoting of the local optical power density of the excitation irradiation, and the LSPR effect on the emission light. In order to compare the enhancement factor on the excitation irradiation, the transition point of the power curves assigned to different film thickness are presented, and implies the film thickness is independent of this effect. Sequentially, they attribute this non-linearly relationship to the coupled frequency of LSPR and emission light, and explain the mechanism via fitting the analytical optical absorption model. The fitting results imply the lower electron density on Au film leads to greater resonance intensity, and a higher enhancement factor on the emission light further. In addition, the component of luminescence carrier determinants the concentration of crystal defect associated with the local crystal field symmetry and fluorescence quenching center. Xing and co-workers have studied the effect of the crystal defect on the luminescence process via different Li/Nb ratio in Ho^3+^/Yb^3+^/Tm^3+^:LN single crystal [44]. They have figured out that the higher Li/Nb ratio decreases the crystal defect and benefits the luminescence process, as shown in Figure 4d. Moreover, the time-resolved fluorescence spectra suggest that the longer intermediate level lifetime and shorter emission level lifetime benefit the luminescence intensity.

#### 5.1.2. Fluorescence Temperature Sensing

Temperature sensing based on the fluorescence characteristic (lifetime, wavelength, intensity, and fluorescence intensity ratio) has attracted great attention due to the feasible applications in nano-scale, high temperature, and extreme environment. Among all these fluorescence temperature sensing strategies, the fluorescence intensity ratio (FIR) technique based on the emission intensities of two corresponding emission levels are widely studied due to the reliable self-reference setting. The main stream of the FIR technique is based on temperature coupled levels (TCLs) wherein electron population densities on the two nearby emission levels (energy gap Δ*E*, 200 cm^−1^ ≤ Δ*E* ≤ 2000 cm^−1^) strictly follow Boltzmann low. Conversely, the sensing coefficient of TCLs strategy is proportional to the energy gap of these two levels and limited by the determiner of Δ*E*. Therefore, great effort has been put forward to promote sensing coefficient, including optimization of luminescence carrier construction, lanthanide ions concentration, and defect levels.

**Figure 5:**
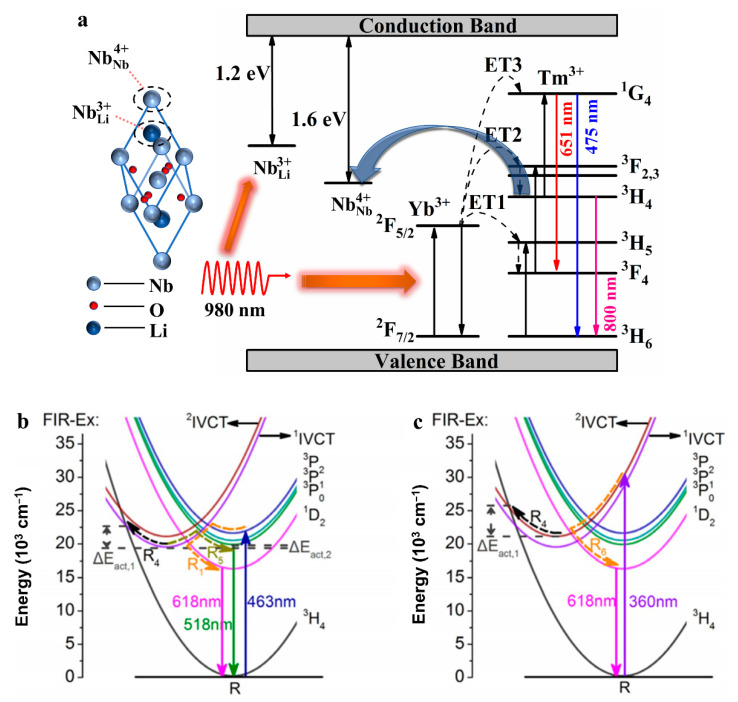
(**a**) The energy level diagram illustrating the luminescence mechanism in Tm^3+^, Yb^3+^: LiNbO_3_ under 980 nm excitation [45]. Schematic configurational coordinate diagrams for the FIR-Ex strategy in Pr:CLN with (**b**) 463 nm excitation and (**c**) 360 nm excitation [46].

Liu and co-workers have grown congruent Tm^3+^/Yb^3+^: LiNbO_3_ single crystal and utilized temperature unstable polaron structure ${Nb}_{Li}^{3+}$ and ${Nb}_{Nb}^{4+}$ to optimize the energy transfer process, as shown in Figure 5a [45]. As a result, the electron populations on the involved two emission levels (Tm^3+^(^3^H_4_) and Tm^3+^(^1^G_4_)) become much more temperature sensitive, giving rise to a defect level modulated fluorescence temperature feedback. Distinguishing from the conventional FIR strategies, Long and co-workers have proposed a novel Ex-FIR strategy based on the FIR of 618 nm emission under 360 and 463 nm irradiation in Pr^3+^:LN single crystal, as shown in Figure 5b,c. Moreover, charge dynamics and energy transfer process are optimized via incorporation of Mg^2+^, which improves the temperature coefficient further. They have also compared the properties of this strategy and conventional FIR strategy, and pointed out this novel strategy presents a much better performance due to the different charge/energy evolution routes under different excitation wavelength [46].

### 5.2. Surface Acoustic-Wave Devices

Radio-frequency (RF) acoustic devices are an essential part of the front ends for emerging applications in 5G and IoT [47,48,49]. In SAW devices, the acoustic wave was propagated along the surface of a piezoelectric material. Periodic metallic bars on a piezoelectric material, called IDT electrodes, were used to excite and receive waves with frequencies of up to several GHz. Thus, the piezoelectric substrate is very important, which need a high electromechanical coupling factor, high quality factor, large acoustic velocity, and low acoustic loss [48]. Among different piezoelectric materials, owing to its electromechanical coupling factor of 5.5%, quality (Q) factor of 10^5^, acoustic velocity of 3400–4000 m/s, high thermal (T_c_ = 1140 °C) and chemical stability, LiNbO_3_ has been served as important piezoelectric substrate for SAW devices [50]. For RF application, different LiNbO_3_ single-crystal cuts have been studied in SAW because that LiNbO_3_ has anisotropic electromechanical coupling factor and acoustic velocity. Recently, one of the most exciting advances is the use of transferred LiNbO_3_ thin films, which were first enabled by the ion slicing technique developed for integrated photonics in the 1990s [49]. The thickness, microstructure of LiNbO_3_ single crystal film have been designed to improve SAW performance in many recent works.

In addition, SAW devices have been served as sensors for using in temperature, pressure measurement. Measuring high temperature with wireless behaviour is significant for using in harsh environmental. LiNO_3_ shows high thermal stability (T_c_ = 1140 °C), thus LiNO_3_ SAW devices are very appropriate for high temperature applications. Duan et al. have reported LiNO_3_ SAW device for wireless sensor application with well temperature dependency [51]. The temperature coefficient of frequency of 16 μm wavelength devices was −87.5 ppm/°C and was −72.41 ppm/°C for 12 μm wavelength devices. Recently, the LiNbO_3_ SAW sensors that can measure up to 1100 °C with a good repeatability and endurance were reported [52]. Distinct linearity of f0 vs. temperature, together with the temperature durability, were verified by conducting various high-temperature RF tests. Such a SAW sensor was attached with an embedded near-field antenna to enhance the wireless transmission ability for future high-temperature remote sensing systems.

**Figure 6:**
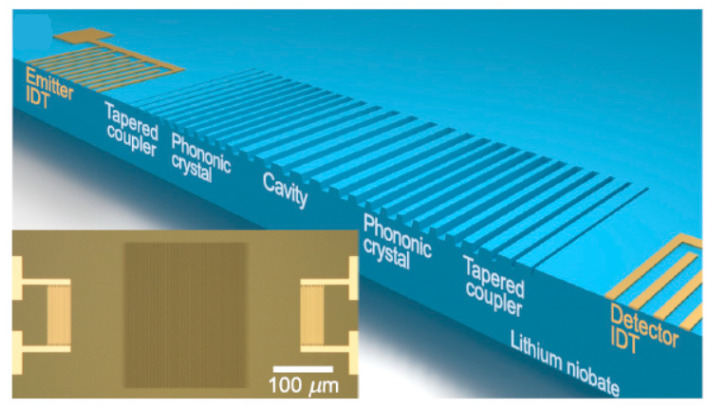
Illustration of band structure engineered surface acoustic resonator on LiNbO_3_. Inset: optical microscope image of a fabricated device. The dark region at the center is the etched grooves, and the bright regions on the sides are metal IDTs [59].

LiNbO_3_ SAW devices also have found their usage in solid state physics, for example, SAW-driven quantized charge transport [53,54], the use of SAWs to control phonon angular momentum [55], the strong optomechanical coupling of individual quantum emitters and a surface acoustic wave [56], and quantum control of surface acoustic-wave phonons [57]. In integrated photonic systems, LiNbO_3_ SAW resonators can be used to confine surface phonons [58]. At GHz frequencies, it is difficult to achieve SAW resonators with a high Q factor and small phonon mode size. Based on Y128° cut LiNbO_3_ crystal, a compact high-Q (6 × 10^4^ at 4 Kelvin) SAW resonators with mode size as low as 1.87 λ^2^ operating at GHz frequencies have been designed (Figure 6) [59]. The f·Q value (>10^13^) and small mode size SAW resonators can be applied in quantum photonics and integrated hybrid systems with phonons, photons, and solid-state qubits. In these applications, LiNbO_3_ SAW were often coupled to other physics system, which demand smart-cut LiNbO_3_ thin film. In future, crystal processing (smart-cut + polishing and wafer bonding) and nanofabrication of LiNbO_3_ single crystal will more and more important in this field.

### 5.3. Electrochemical Applications

**Figure 7:**
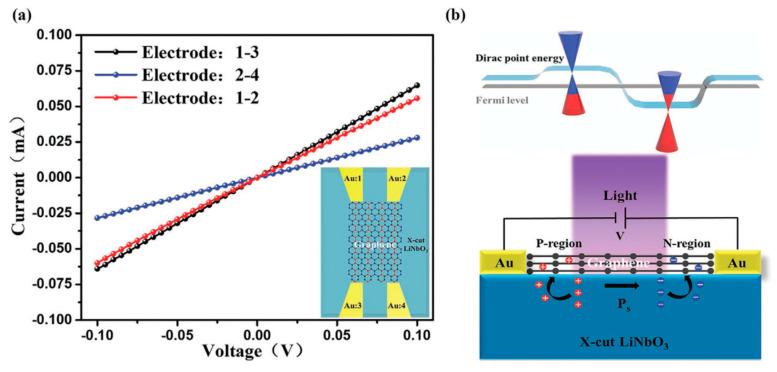
(**a**) The current–voltage (I–V) curve of the sample in the same environment. The black, blue, and red color lines represent measurements between electrodes 1–3, electrodes 2–4, and electrodes 1–2, respectively (cf. inset). (**b**) Band diagrams of the device at the p−n junction state [62].

**Figure 8:**
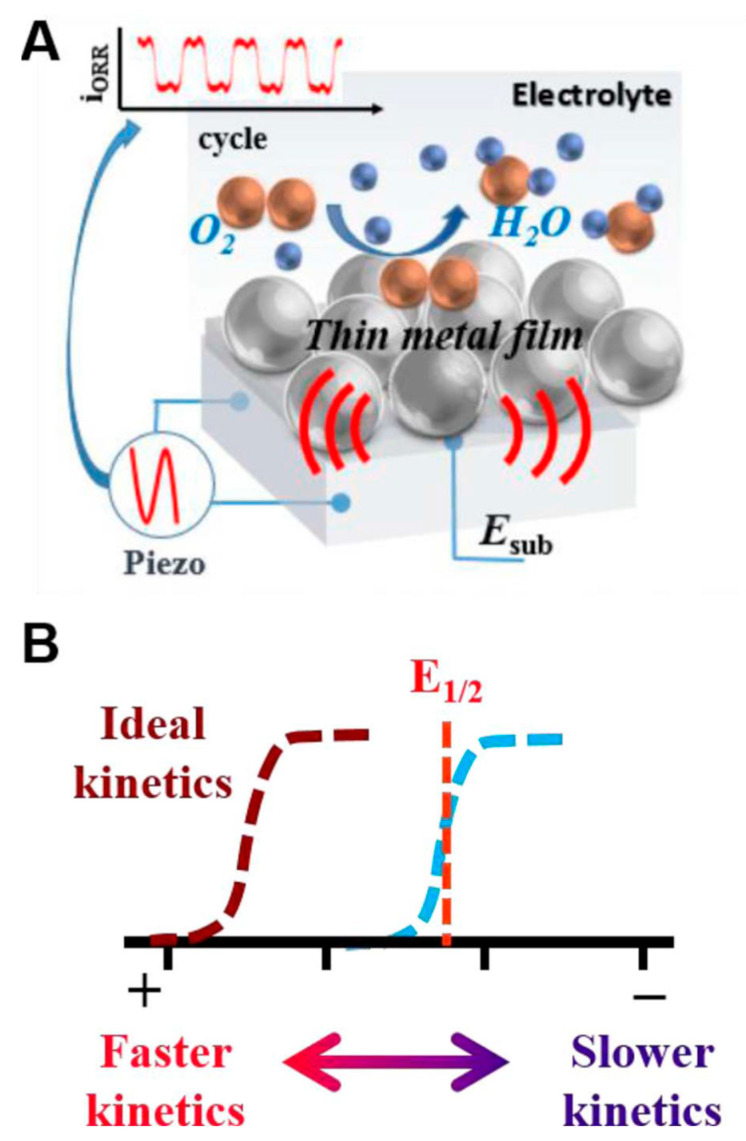
Action of a piezoelectric substrate on the reactivity of the ORR on platinum. (**A**) Piezoelectric control of platinum strain determines oxygen reduction reactivity. (**B**) Theoretical steady state voltammetric response of the ORR when changing the reaction kinetics with piezoelectric actuation. Shifts toward more positive potentials indicate increased kinetics, while shifts toward negative potentials indicate more decreased kinetics. The ideal ORR wave would have its half-wave potential at 1.23 V vs. NHE [64].

LiNbO_3_ has the advantages of spontaneous ferroelectric polarization, high dielectric constant, high chemical stability, and high voltage electric coefficient, which can be served as substrate to improve photodetector, catalysis, photoreactivity, and battery performances of other materials [60,61]. LiNbO_3_ polarized doping has been used to enhance the photoelectric detection characteristics of graphene [62]. As shown in Figure 7, the devices were fabricated with graphene deposited on x-cut LiNbO_3_ bulk and film crystals. The local ferroelectric polarization of x-cut LiNbO_3_ leads to the formation of n- and p-doping of graphene at the same time. This p–n junction photodetector shows a wide detection range of 405 to 2000 nm, a responsivity of ≈2.92 × 10^6^ A/W at an incident power of 24 pW (λ = 1064 nm), a high detectivity of ≈8.65 × 10^14^ Jones, and a fast rise/decay time of ≈23 ms/≈23 ms. The oxygen reduction reaction (ORR) is a key reaction for fuel cell, biological electrocatalysis, and air-batteries [63]. It is difficult to achieve ORR reaction at the standard potential of 1.23 V vs. NHE. A piezoelectric electroanalytical platform for modulating ORR reactivity has been formed with a thin layer of Pt deposited on LiNbO_3_ single crystal substrate (Figure 8) [64]. Piezoelectric actuation caused up to a ∼10 mV positive shift for the ORR reduction wave, when compared to curves in the absence of actuation.

The photoreactivities have been also enhanced by spontaneous polarization of LiNbO_3_ single crystal [65]. In photocatalytic water reduction, c+ LiNbO_3_ was superior to c− LiNbO_3_, while c− LiNbO_3_ exhibited better performances for photoelectrochemical water oxidation than c+ LiNbO_3_ [66]. The results shows that c− LiNbO_3_ can favor the hole transport from the bulk to the surface compared with c+ LiNbO_3_, leading to the anisotropic performances of c+ and c− LiNbO_3_ in water oxidation/reduction. Thus, tuning the LiNbO_3_ polarization direction may be a novel method to enhance the photoreactivities of water oxidation or reduction.

**Figure 9:**
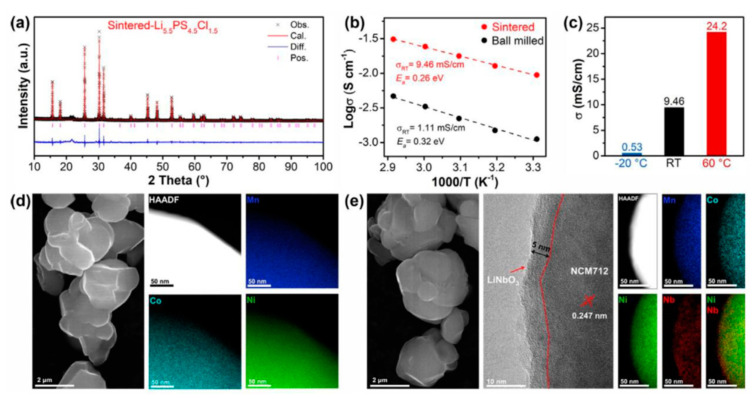
Characterizations of LiNbO_3_-coated LiNi_0.7_Co_0.1_Mn_0.2_O_2_ (LNO@NCM712) [70]. (**a**) XRD Rietveld refinement of the prepared Li 5.5 PS 4.5 Cl 1.5. (**b**) Arrhenius plots of the Li 5.5 PS 4.5 Cl 1.5 electrolytes obtained from milling and annealing processes. (**c**) The ionic conductivities of the annealed Li 5.5 PS 4.5 Cl 1.5 electrolyte at different temperatures (−20 °C, RT, and 60 °C). SEM/TEM images and the corresponding EDX mapping results of (**d**) the pristine NCM712 and (**e**) LNO@NCM712.

Surface modification of high voltage cathodes is important method to improve electrochemical performance of Li-ion battery [67,68,69]. LiNbO_3_-coating has been found to be a useful strategy to improve stability of electrode materials [68,69]. Recently, the electrochemical behavior of LiNbO_3_ coating on LiNi_0.7_Co_0.1_Mn_0.2_O_2_ electrode (LNO@NCM712) have been studied (Figure 9) [70]. The LNO@NCM712 electrode delivers initial discharge capacities of 80.9 and 138.9 mAh/g at 5 C under RT and 60 °C respectively. LNO@NCM712 shows capacity retentions of 87.5% and 88% after 600 and 300 cycles, which were better than that of the NCM712 electrode. The results show that LNO@NCM712 can mitigate volume changes during cycling and reduce side reaction between solid electrolytes and active materials. The performance enhancement is owing to the high chemical stability and Li diffusion of LiNbO_3_.

### 5.4. Domain Engineering, Memristors, Sensors, and Harvesters

Thanks to its ferroelectric and piezoelectric properties, LiNbO_3_ crystal can also applied in critical modern areas as sensors, harvesters, and memristors.

Domain engineering—LiNbO_3_ is a well-known uniaxial ferroelectric materials, which displays spontaneous polarization aligned along the Z-axis. There exist many domains in LiNbO_3_ single crystals. Domain engineering plays a pivotal role in the development of nonvolatile transistors, domain wall (DW) memory devices. Kampfe et al. had tracked head-to-head charged domain walls (CDWs) across millimeter-thick LiNbO_3_ single crystal by Cherenkov second-harmonic generation [71]. Furthermore, tip-induced polarization reversal on a nonpolar cut of LiNbO_3_ single crystal has been proved, and complex domain structures consisting of a few separate domains were found [72]. Many different methods have been designed to control LiNbO_3_ domain engineering for applying in data computing, storage, and sensing operations. The stabilized the head-to-head DWs, neutral DWs, and tail-to-tail DWs within a LiNbO_3_ transistor have been achieved by controlling charge injection in compensation of the domain boundary charge under applied drain–gate, drain–source, and gate–source voltages [73].

Memristors—Application of conducting ferroelectric DWs as functional elements may facilitate development of conceptually new resistive switching devices, memristors. To reduce coercive voltage of LiNbO_3_, Chaudhary et al. had used Pt/LiNbO_3_/graphene capacitors to perform resistance modulation [74]. The resistance of memristor device set to a polydomain state can be continuously tuned by application of subcoercive voltage. The tuning mechanism was based on the reversible transition between the conducting and insulating states of DWs. The curved wall region located near the domain tip that connects the two top nanoelectrodes exhibiting 12-fold magnification of the on-current when compared with the straight wall region near the tail. Recently, highly curved DWs that were exposed at the LiNbO_3_ film surface in high conduction have been created [75]. To improve the polarization retention in LiNbO_3_ single crystal memory cells, Jiang et al. have etched X-cut LiNbO_3_ crystals to form the etching angles (h) as high as 83° [76]. LiNbO_3_ crystal also has been used in high temperature ferroelectric domain wall memory, which showed good retention of written information with a large on/off current ratio of ~104 at 450 K [77].

Sensors—Owing to its piezoelectric properties, LiNbO_3_ can be applied in some sensors. Bidomain LiNbO_3_ crystal has been served as substrate to detect ultra-weak low-frequency vibrations. The smallest detectable vibration was 0.1 nm at frequencies above 38 Hz [78]. Its sensitivity varied from minimum values of 20 μV/nm and 7 V/g (where g = 9.81 m/s^2^ is the gravitational acceleration), at a frequency of 23 Hz, to peak values of 92.5 mV/nm and 2443 V/g, at the mechanical resonance of the cantilever at 97.25 Hz. Sensitive magnetic sensors that can detect very weak magnetic fields with amplitudes lower than 10 pT, and frequencies below 100 Hz are very important for biomedical applications, such as magnetoencephalography and magnetocardiography. The magnetic sensor, made of magnetoelectric bi-layered long bar composite formed by a thin top metglas layer and a bottom bidomain LiNbO_3_ single crystal, can display large voltages in response to weak magnetic fields [79]. The magnetoelectric coefficients was as large as 550 V (cm·Oe)^−^^1^, corresponding to a conversion ratio of 27.5 V Oe^−^^1^, under resonance conditions at frequencies of the order of 100 Hz in magnetic bias fields as low as 2 Oe. Equivalent magnetic noise spectral densities were down to 120 pT Hz^−1/2^ at 10 Hz and 68 pT Hz^−1/2^ at 81 Hz.

Harvesters—Self-powered electrical microsystems that are capable of harvesting locally available forms of energy would avoid the need for batteries especially in health monitoring systems. Low-level ambient vibrations provide a ubiquitous source of ambient energy. Thanks to LiNbO_3_ for having one of the largest transversal voltage *g*_31_ constants among all piezoelectrics of up to ca. 35.6 mV m/N, *k*31 up to 0.52, and *Q* factors on the order of 1000, as well as dielectric losses of less than 1%, LiNbO_3_ is also proper applied in vibrational energy harvester. With the use of bidomain Y128-cut LiNbO_3_ crystals, the harvester yielded an open-circuit voltage of 1.54 kV/g at a low bending resonance frequency of ca. 32.2 Hz and output power density of up to 11.0 mW/(cm^3^·g^2^) [80]. Another harvester with a flexible beam of 65 mm length and a tip mass made of a LiNbO_3_ thick film bonded on silicon produced power density of 965 μW/cm^2^/g^2^, which is among the highest reported values compared to both Pb- and Pb-free vibrational harvesting devices [81].

## 6. Conclusions and Outlook

LiNbO_3_ materials are one important kind of multiple functionality, which can be applied in optical waveguide, illumination, photodetector, sensors, laser, SAW, SHG, energy-related devices (Li-ion battery, electrolysis), memristors, harvesters, and quantum-related solid state physics. In 2017, researchers at Harvard University developed a technique to fabricate high-performance optical microstructures using LiNbO_3_, which opens the door towards a variety of intriguing functionalities, enabled by the unique optical and electrical properties of LiNbO_3_ that do not exist in in other optical media [82]. This research demonstrates that this relatively unexplored material has been ready to address critical applications in optical links for data centers. With the development of smart cut, nanofabrication technologies, thin-film LiNbO_3_ and LiNbO_3_ on insulator (LNOI) have been developed to enable the construction of tiny, inexpensive, low-power devices. Recently, LiNbO_3_ has found its many studies used in integrated photonic circuits, quantum photonics, microwave-to-optical conversion, and more. In addition, some traditional devices have found new application areas. For example, serving as one important RF part, SAW devices have found new application in high-temperature sensors, wireless technology, quantum-related subjects, and solid-state physics.

Structure–performance relationship is an important goal in the materials field. To demand new application requirements of LiNbO_3_, crystal and defect structures, crystallization, and characterization must be revisited. Crystal and defect structures of LiNbO_3_ are the origin of its functionality. With the help of advanced characterization and calculation tools, some debates need to be proofed, such as site defect types, defect cluster, domain, Li content, and more.

## Figures and Tables

**Table 1:** Growth parameters of LiNbO_3_ reported in literatures.

Pulling Rate (mm/h)	Rotation Rate (rpm)	Size ϕ × l (mm)	Li Content (mol%/cm)	Ref.
1–2	5–10	153 × 110	Δ[Li_2_O] ≈ 0.001	[26]
0.3–3	20–35	8 × 10	-	[27]
1	7	30 × 50	-	[28]
0.4–1.5	10–30	50 × 30	Δ[Li_2_O] < 0.005	[29]
1–2.5	10–25	80 × 60	Δ[Li_2_O] < 0.02	[30]
2.8–4.0	3–10	100 × 80	Δ[Li_2_O] < 0.002	[22]

**Table 2:** Testing method of Li composition for LiNbO_3_.

Testing Method	Advantages	Disadvantages
Raman scattering method	Raman systems have become cheaper and easier to use	The use of a correct configuration of the detection and excitation polarizers (in the case of single crystals)
Curie temperature	Linearly with Li/[Li + Nb] ratio Reliable and sufficient sensitivity for composition	High Curie temperature close to the melting point
UV absorption edge	Convenient and accurate way for determining the composition	Nonlinear relationship Accuracy is governed by the wavelength calibration Doping compound will deteriorate the accuracy
Refractive indices	Function of wavelength and stoichiometry	Nonlinear relationship
Birefringence	Approximately linear correspondence between Li content and birefringence	The nonlinear relationships dominated by the wavelength

## Sample Availability

Samples are available from the authors.
